# Cystic Adventitial Disease in Former Athlete

**DOI:** 10.3889/oamjms.2015.084

**Published:** 2015-07-09

**Authors:** Nikola Fatic, Aleksandar Nikolic, Dejan Maras, Nikola Bulatovic

**Affiliations:** *Clinical Centre of Montenegro, University of Montenegro, Podgorica, Montenegro*

**Keywords:** cystic adventitial disease, popliteal artery, intermittent claudication

## Abstract

In this paper we present a 39-year old former athlete complaining with pain in his legs during long walk resembling to intermittent claudication. Color duplex scan described a popliteal artery with 10 mm in diameter with mural thrombus that caused stenosis 75% of lumen. Digital subtraction angiography demonstrated a stenosis of right popliteal artery. The suspicion for Cystic adventitial disease was set. The patient was operated on by posterior direct approach. After incision, a yellowish viscous material was observed in adventitia. Partial resection of the affected popliteal artery and replacement by an autogenous great saphenous vein graft was performed. Patient was dismissed on the seventh postoperative day, in good condition and without any complication.

Cystic adventitial disease of the popliteal artery should be considered in the differential diagnosis of intermittent claudication, especially in former sportsmen patients. Partial resection of the affected popliteal artery and replacement by an autogenous great saphenous vain graft produces excellent results.

## Introduction

Cystic adventitial disease (CAD) is rare vascular disease characterized by mucin containing cyst in the adventitial layer of the artery [1]. Atkins at all, reported the first case of CAD in 1947, in a young patient caused by cystic deformation of the external iliac artery [2]. CAD mostly affected healthy middle-aged males, who do not have risk factors for atherosclerotic disease [3], with a male-to-female ratio of 15:1. It is assessed to occur in one out of every 1,200 of individuals suffering from claudication [4]. The condition is predominantly located in the popliteal artery (approximately **85%** of cases) [5]. Compression of the arterial lumen by cists can cause intermittent claudication.

## Case Report

**Figure 1:**
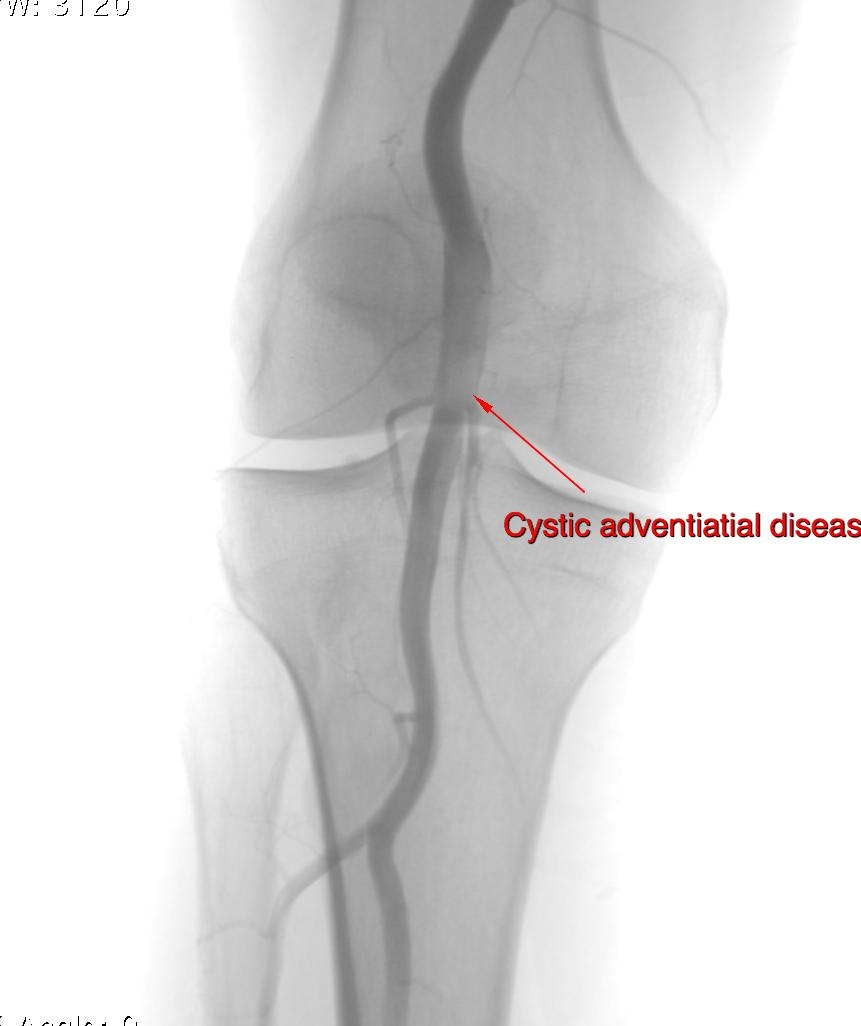
*Digital subtraction angiography of popliteal artery*.

**Figure 2:**
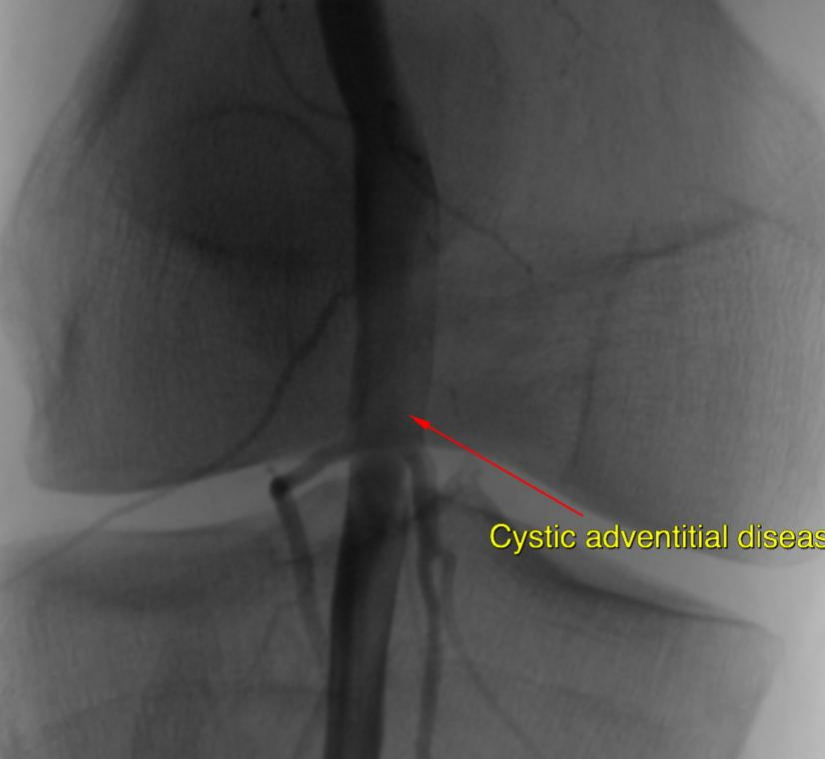
*Digital subtraction angiography of popliteal artery with cystic adventitial disease in focus*.

A 39-year-old man former athlete was investigated for pain in the right popliteal fossa and lower leg caused by walking. The claudication distance was about fifty meters. The symptoms have started suddenly, approximately one month prior to hospitalization. The recovery time after running was sometimes about 10 min. Vascular risk factors were not reported. There was not history of trauma. The lower extremities didn’t reveal signs of ischemia and all the palpable pulses were present, but pulses were weaker as compared to the contralateral limb. Color duplex scan (CDS) described a popliteal artery with 10 mm in diameter with mural thrombus that caused stenosis 75% of lumen. Digital subtraction angiography (DSA) demonstrated a stenosis of right popliteal artery (Figure 1 and 2).

**Figure 3:**
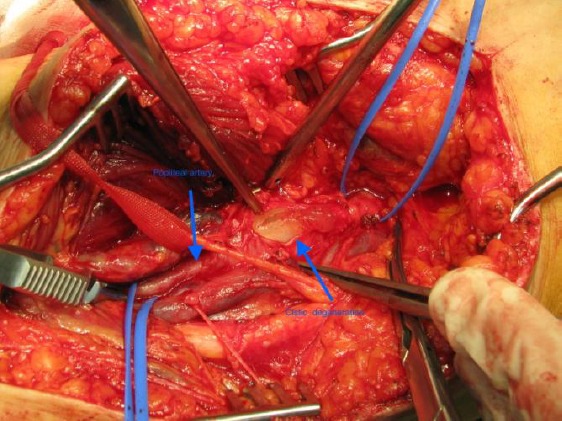
*Intraoperative finding of cystic adventitial disease*.

The suspicion for CAD was set. The patient was operated on by posterior direct approach. After incision, a yellowish viscous material was observed in adventitia (Figure 3). Partial resection of the affected popliteal artery and replacement by an autogenous great saphenous vein graft was performed. The pathological examination revealed cystic adventitial disease of the popliteal artery.

In the postoperative period the patient received acetylsalicylic acid as antiplatelet agent. Patient was dismissed on the seventh postoperative day, in good condition and without any complication. Control CDS at discharge, after first and third month confirmed patent findings.

## Discussion

Cystic adventitial disease (CAD) is rare vascular disease characterized by mucin containing cyst in the adventitial layer of the artery [1]. CAD mostly affected healthy middle-aged males, who do not have risk factors for atherosclerotic disease [3].

CAD is predominantly situated in the popliteal artery (approximately 85 % of all cases) but other authors have reported affection of the other localizations (external iliac, common femoral, axillary, distal brachia, radial and ulnar arteries, the common femoral, popliteal or the great saphenous vein) [5]. Considering histopathology, there are different types of cysts with viscous material consisted of proteoglycans, mucopolysaccharides and mucoproteins, in addition to hyaluronic acid [4]. There are lots of theories about the causes of cyst’s developing. One of the theories hypotize that repeated micro trauma of vessels close to joints cause cystic degeneration of the adventitia [6-11]. It may explain CAD in our patent (Repeated trauma during the training caused micro trauma and cystic degeneration of the adventitia).

The similar symptoms as a peripheral arterial disease and popliteal entrapment syndrome make differential diagnosis of the CAD of the popliteal artery very difficult. The cysts may compress the artery during muscle activity and flexion of the knee. That can be explanation of the pain reported by our patient. In the neutral position arterial pulses can be normal but they are less intense or disappear with knee flexion. In the present case, the popliteal and pedal pulses in the affected leg were weaker as compared to the contralateral limb, due to a stenotic arterial lesion visualized in the CDS and DSA.

Ultrasonography and CDS may demonstrate lesions within the affected arterial wall. DSA may show curvilinear/spiral narrowing of vessels with a paucity of collaterals and an absence of post-stenotic dilatation [12]. Other imaging modalities have also been useful to diagnose CAD such as Computed tomography (CTA) and magnetic resonance imaging angiography (MRA) [13, 14].

The irregularities and stenosis of the vessel lumen lead to unsure diagnosis. In our patient, age, symptoms, CDA and DSA was enough to set suspicion of CAD.

Spontaneous resolution of CAD has been described [4]. Timely set diagnosis and clever choice of treatment can prevent complications (thrombosis of the popliteal artery and distal thromembolisation). Three treatment options are currently advised for CAD: resection of the cyst with arterial preservation, excision of the involved arterial segment with interposition bypass grafting, and CT- or ultrasound-guided percutaneous cyst aspiration. The treatment of choice is surgical removal of the cyst, which achieves complete excision of the cyst wall and preserves the medial and intimal layers. This approach is most suitable for patients without severe popliteal stenosis and minimal adherence between the cyst wall and the artery [4]. Cases of severe arterial stenosis or occlusion necessitate interposition bypass grafting, especially when extrinsic compression has been released [4]. This observation leads us to perform resection of the affected popliteal artery and replacement by an autogenous great saphenous vain graft. Although ultrasound- or CT-guided aspiration has been used successfully to treat CAD of the popliteal artery, the reported frequency of recurrence after aspiration is high. There is one report of endovascular stenting being used to treat popliteal artery stenosis that arose secondary to CAD with significant claudication. However, despite technical success, the implanted stent remained patent for only 1 week and the patient eventually underwent interposition venous graft reconstruction to resolve the CAD [1].

In conclusion, CAD of the popliteal artery should be considered in the differential diagnosis of intermittent claudication, especially in former sportsmen patents. Ultrasound and DSA can be useful tools for setting diagnosis of CAD. Partial resection of the affected popliteal artery and replacement by an autogenous great saphenous vain graft produces excellent results.
